# Characterization of Secondary Bacterial Infections and Antibiotic Use in Mechanically Ventilated Patients With COVID-19 Induced Acute Respiratory Distress Syndrome

**DOI:** 10.1177/08850666211021745

**Published:** 2021-08-10

**Authors:** Erik Risa, David Roach, Jehan Z. Budak, Christopher Hebert, Jeannie D. Chan, Nandita S. Mani, Chloe Bryson-Cahn, James Town, Nicholas J. Johnson

**Affiliations:** 1 University of Washington School of Medicine, Seattle, WA, USA; 2 Division of Pulmonary, Critical Care, and Sleep Medicine, University of Washington School of Medicine, Seattle, WA, USA; 3 Division of Allergy and Infectious Diseases, University of Washington School of Medicine, Seattle, WA, USA; 4 Harborview Medical Center, University of Washington School of Pharmacy, Seattle, WA, USA; 5 Department of Emergency Medicine, University of Washington School of Medicine, Seattle, WA, USA

**Keywords:** COVID-19, mechanical ventilation, nosocomial infections, antibiotic use, ARDS

## Abstract

**Background:**

COVID-19 has a widely variable clinical syndrome that is difficult to distinguish from bacterial sepsis, leading to high rates of antibiotic use. Early studies indicate low rates of secondary bacterial infections (SBIs) but have included heterogeneous patient populations. Here, we catalogue all SBIs and antibiotic prescription practices in a population of mechanically ventilated patients with COVID-19 induced acute respiratory distress syndrome (ARDS).

**Methods:**

This was a retrospective cohort study of all patients with COVID-19 ARDS requiring mechanical ventilation from 3 Seattle, Washington hospitals in 2020. Data were obtained via electronic and manual review of the electronic medical record. We report the incidence and site of SBIs, mortality, and antibiotics per day using descriptive statistics.

**Results:**

We identified 126 patients with COVID-19 induced ARDS during the study period. Of these patients, 61% developed clinical infection confirmed by bacterial culture. Ventilator associated pneumonia was confirmed in 55% of patients, bacteremia in 20%, and urinary tract infection (UTI) in 17%. *Staphylococcus aureus* was the most commonly isolated bacterial species. A total of 97% of patients received antibiotics during their hospitalization, and patients received nearly one antibiotic per day during their hospital stay.

**Conclusions:**

Mechanically ventilated patients with COVID-19 induced ARDS are at high risk for secondary bacterial infections and have extensive antibiotic exposure.

## Introduction

The SARS-CoV-2 pandemic has led to a global surge in the utilization of intensive care and mechanical ventilation.^1^ Previous experiences with other respiratory viral pandemics, most notably H1N1 influenza, demonstrate a high burden of secondary bacterial infections (SBIs) that occur in critically ill patients receiving mechanical ventilation.^2^ These SBIs account for a significant increased morbidity, intensive care unit (ICU) length of stay, and mortality.^3^ Given the recent emergence of SARS-CoV-2, the characterization and extent of SBIs in critically ill patients with COVID-19 has not been as thoroughly documented in the literature, leading to antimicrobial stewardship guidelines in patients with COVID-19 that rely on emerging evidence.^4,5^ These factors create challenges surrounding the appropriate use of antibiotics in mechanically ventilated patients with COVID-19, possibly contributing to their overuse and a subsequent increase in antimicrobial resistance.^6–8^

COVID-19 pneumonia causes a clinical syndrome that is often difficult to distinguish from a community-acquired bacterial pneumonia, leading to empiric antibiotic use despite low levels of co-infection on initial presentation.^9,10^ In addition, hospitalized patients with COVID-19 frequently have a persistent inflammatory syndrome that has overlapping clinical features with bacterial sepsis, resulting in high utilization of antimicrobial therapy in these populations despite reports of overall low prevalence of SBIs.^11,12^ However, it is well known that there are higher rates of SBIs in critically ill patients who are on mechanical ventilation, although studies looking at this specific population in COVID-19 are lacking.^11,13^ Given the complicated balance of providing adequate antibiotic therapy while practicing antimicrobial stewardship, there have been calls for more data to guide antimicrobial stewardship efforts.^14–16^

In this study we describe the incidence, source, and bacterial species of all SBIs in mechanically ventilated patients with COVID-19 across 3 hospitals in Seattle, WA. We additionally describe the antibiotic regimens and days of therapy for these patients during their hospital admission, with the goal to elucidate the patterns of antibiotic prescribing.

## Methods

### Setting and Study Design

This is a multicenter, retrospective cohort study using data extracted from the electronic health record at 3 hospitals within a single health system between January 1, 2020 and December 31, 2020. This study was approved by the University of Washington Human Subjects Division (STUDY00011469). Two of the hospital sites, Harborview Medical Center (HMC) and the University of Washington Medical Center (UWMC)—Montlake Campus, are tertiary care facilities that serve as regional referral centers for extracorporeal life support (ECLS). The third facility, UWMC—Northwest Campus, is a community hospital. All patients who were admitted to the ICU, underwent mechanical ventilation, and had positive SARS-CoV-2 PCR tests by either nasopharyngeal swab or ET aspirate were included. Data were extracted electronically from the medical record. Patient charts were then manually reviewed by either a critical care or infectious diseases physician. If patients were Sars-CoV-2 positive and required mechanical ventilation due to respiratory failure from COVID-19 pneumonia, they were included for further review, while patients were excluded who were Sars-CoV-2 positive but underwent mechanical ventilation for an alternative reason (for example, stroke or trauma).

### Bacterial Culture Data

All bacterial cultures collected during routine clinical care were reviewed. Positive cultures that occurred within the first 48 hours after hospitalization were excluded to capture only nosocomial infections. Blood cultures growing normal skin flora (for example, Coagulase-negative staphylococci [CoNS], diptheroids) were only included if they were drawn from 2 separate sites concordantly or in consecutive days. Urine cultures had to meet a threshold of >100,000 colony forming units per milliliter for inclusion, and were further assessed for meeting National Healthcare Safety Network (NHSN) criteria.^17^ Respiratory cultures growing normal oral flora, “mixed flora,” and yeast were excluded, as were isolates which were not speciated out (for example, “GNRs”). *Clostridioides difficile* was identified by polymerase chain react (PCR) Xpert CDI Epi assay.

Isolates were considered to be “unique” infections for each new body compartment from which they were isolated. In many patients, for example, a given species was repeatedly sampled from the lower respiratory tract over the course of the hospital stay, but all isolates were considered to be a single unique infection. However, if the same organism was isolated from both the lower respiratory tract and the blood, this was considered as 2 unique infections. Isolates that were sampled from sputum, bronchoalveolar lavage, or tracheal aspiration were all considered as “lower respiratory cultures.” Patients were considered to have a VAP if they had lower respiratory cultures growing bacteria meeting the inclusion criteria above and underwent treatment for a VAP by their clinical care teams.

### Antibiotic Usage

To collect data on antibiotic usage, the electronic medical record for each patient was manually reviewed, and for each antibiotic, any single day that an antibiotic dose was administered was included as an antibiotic day of therapy (DOT). If only a single dose were administered of an antibiotic that is normally dosed multiple times daily, this was considered as one DOT. Conversely, for antibiotics with long half-lives such as vancomycin, only the days in which the antibiotic dose was administered were tallied. Choice and duration of antimicrobial therapy was determined by the clinical teams providing direct care to the patients.

### Statistical Methods

Univariate statistics including frequency counts and percentages were used to describe the baseline characteristics of the study population. Chi-square significance testing was performed to test association between the development of an SBI and patient outcomes. Calculations and generation of figures were conducted with R version 4.0.0 and the tidyverse package.^18^

## Results

### Patient Population and Clinical Characteristics

**Figure 1.**
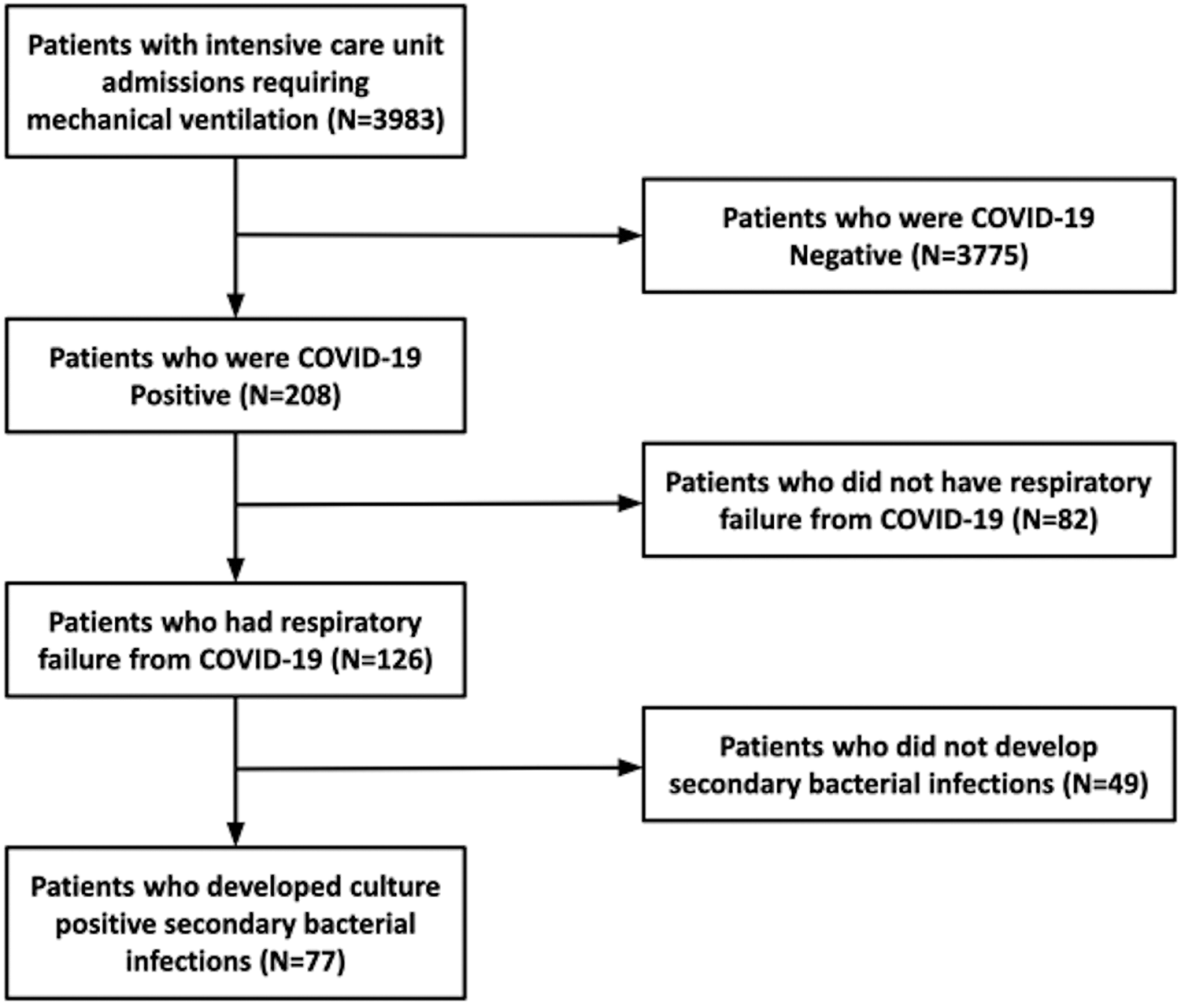
Flow chart demonstrating patient selection criteria.

Over the course of the study period, 208 SARS-CoV-2 patients with COVID-19 were admitted to the ICU and required mechanical ventilation. After review of the patient clinical records, 82individuals either required mechanical ventilation for less than 48 hours or for reasons other than respiratory failure and were not included in further analysis. The final sample population included a total of 126 patients with respiratory failure requiring mechanical ventilation secondary to COVID-19 (Figure 1). Sixty-eight (54%) were hospitalized at Harborview Medical Center, 34 (27%) at the UWMC-Montlake, and 24 (19%) at UWMC-Northwest Hospital. The mean patient age was 59 (±15) years, and 88 patients (70%) were male. The most common comorbidities documented were hypertension (45%), diabetes (45%), and atrial fibrillation (19%). The mean duration of ICU care was 16.7 days (±17.5) with 15.8 days (±14.6) spent receiving mechanical ventilation. In total, 107 (84%) patients required vasopressors during their stay, 92 (72%) underwent neuromuscular blockade, 21 (16%) were placed on extracorporeal membrane oxygenation, and 50 (39%) underwent prone positioning at least once during their stay. During the study period, a total of 57 (45%) patients died. Patients who contracted SBIs had a higher mortality (37/77, 48%) compared to those who did not contract SBIs (20/49, 40.8%) but this number did not reach statistical significance (*P* = 0.34).

### Secondary Bacterial Infections

**Figure 2.**
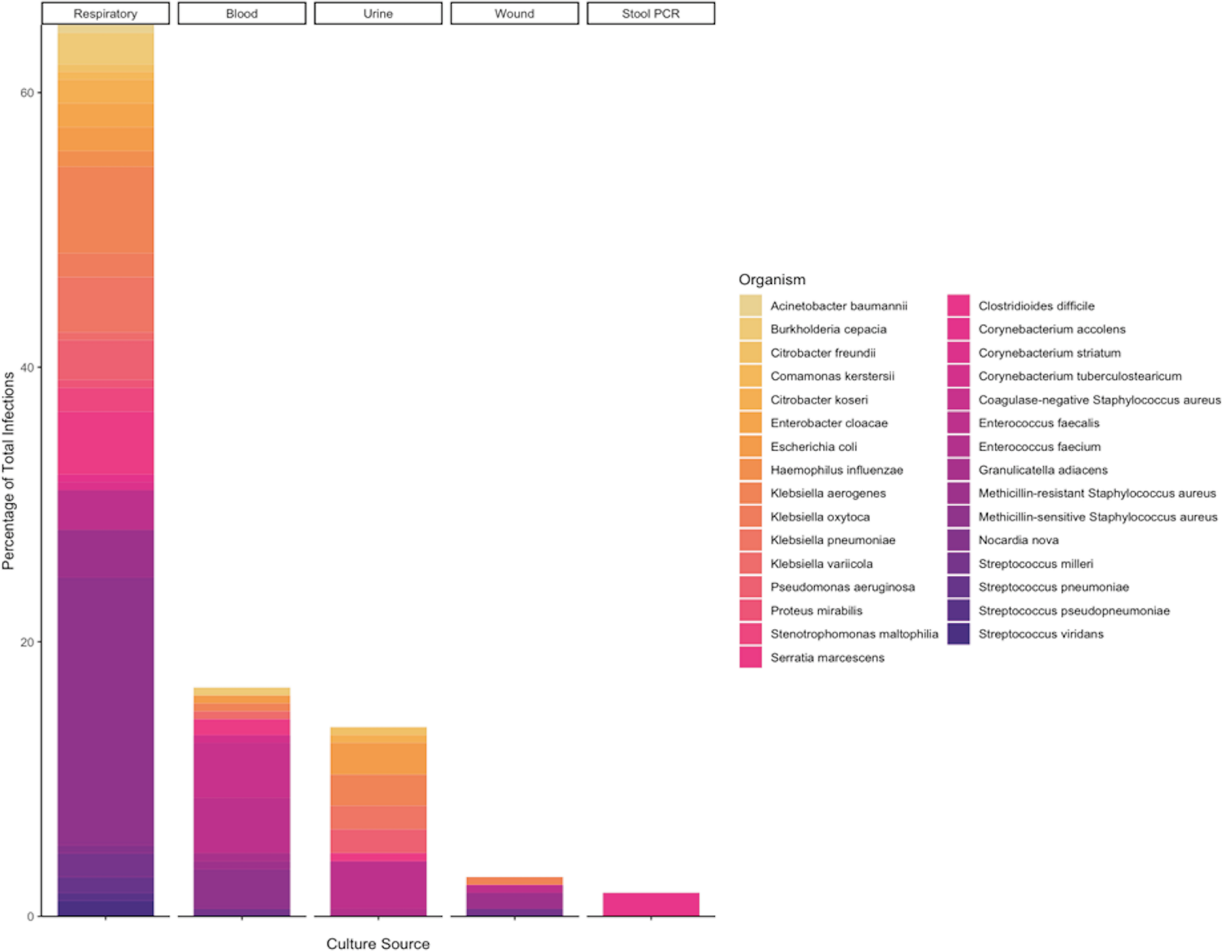
Bar chart demonstrating each type of infection (x-axis) as a fraction of total infections (y-axis). The different colors in the bar chart are proportional to the bacterial species isolated from each source. Gram-negative bacteria are listed in the left column and are represented in gold tones while gram-positive bacteria are in the right column and are colored in purple tones.

Of the 126 patients included in this analysis, 77 (61%) had positive bacterial cultures that met inclusion criteria, resulting in a total of 174 unique infections. Forty-eight patients were transferred from outside facilities, and culture data for their initial 48 hours of hospitalization were unavailable. Of the remaining 78 patients, 10 (12.8%) had positive bacterial cultures within the first 48 hours of admission, consistent with community acquired infection, and these infections were not included in further analysis of the SBIs (Online Appendix A).Only 10 infections were recorded prior to patients undergoing intubation, and the median time to first positive culture after intubation was 9.5 days (IQR, 2.9-18.4). A total of 31 different species were represented (Table 3). The most common bacterial species was *Staphylococcus aureus*, which accounted for 48 (28%) infections. Thirty-nine (81%) of these infections were caused by methicillin-sensitive *S. aureus* (MSSA) while 9 (19%) were caused by methicillin-resistant *S. aureus* (MRSA). The second most common organism was *Enterococcus faecalis* (19 infections), followed by *Klebsiella aerogenes* (17 infections) and *Serratia marcescens* (11 infections) (Figure 2).

**Table 1.** Demographics.

	Overall (N = 126)
Age	
Mean (SD)	59 (± 15)
Sex	
M	88 (70%)
BMI	
Mean (SD)	32 (± 9.1)
Race	
American Indian or Alaska Native	3 (2%)
Asian	19 (15%)
Black or African American	11 (9%)
Multiple races	1 (1%)
Native Hawaiian or Other Pacific Islander	2 (2%)
Unknown	18 (14%)
White	72 (57%)
Ethnicity	
Hispanic or Latino	43 (34%)
Not Hispanic or Latino	66 (52%)
Unavailable or Unknown	17 (13%)
Facility	
Harborview Medical Center	68 (54%)
U. of Washington Medical Center—Northwest Campus	24 (19%)
U. of Washington Medical Center—Montlake Campus	34 (27%)
Past medical history	
Hypertension	57 (45%)
Diabetes	56 (45%)
Atrial fibrillation	24 (19%)
Coronary artery disease	22 (17%)
Heart failure	16 (13%)
End stage renal disease	14 (11%)
Chronic obstructive pulmonary disease	7 (6%)
History of cerebrovascular accident	2 (2%)

**Table 2.** Baseline Characteristics and Clinical Outcomes Comparing Patients With SBIs to Those Without.

	Secondary bacterial infection (N = 77)	No secondary bacterial infection (N = 49)	*P*-Value
Baseline characteristics and measures of illness severity			
BMI	31.4 (8.03)	32.6 (10.9)	0.54
SOFA first 24 hours	9.43 (4.33)	8.10 (4.62)	0.19
SOFA highest during hospitalization	13.8 (2.63)	11.9 (3.35)	0.011
Durations			
Time to first positive culture (days)	11.5 (± 9.5)		
Duration of ICU care (days)	23.5 (19.4)	6.54 (6.21)	<0.001
Hospital length of stay (days)	31.6 (21.6)	15.5 (8.82)	<0.001
Duration of mechanical ventilation (days)	21.8 (16.4)	7.63 (5.58)	<0.001
Duration of prone positioning (hours)	119 (203)	62.8 (155)	0.084
Time to peak respiratory deterioration (hours)	28.5 (72.1)	30.4 (71.5)	0.90
Central line duration (days)	25.3 (23.8)	5.78 (8.44)	<0.001
Therapeutic interventions			
Neuromuscular blockade	57 (74.0%)	33 (67.3%)	0.21
Reintubation rate	26 (33.7%)	7 (14.3%)	0.015
Required hemodialysis	13 (16.8%)	5 (10.2%)	0.35
Required ECLS	19 (24.6%)	2 (4.1%)	<0.001
Received prone positioning	37 (48.0%)	12 (24.5%)	0.0056
Required vasopressors	65 (84.4%)	40 (81.6%)	0.27
Clinical outcomes			
Mortality	37 (48.0%)	20 (40.8%)	0.34

Abbreviations: BMI, body mass index; CAM, confusion assessment method; SOFA, Sequential Organ Failure Assessment; ECLS, extracorporeal life support.

**Table 3.** Organisms and Culture Source.

Organism	Respiratory (N = 113)	Blood (N = 29)	Urine (N = 24)	Wound (N = 5)	Stool PCR (N = 3)	Overall (N = 174)
Acinetobacter baumannii	1 (1%)	-	-	-	-	1 (1%)
Burkholderia cepacia	4 (4%)	1 (3%)	-	-	-	5 (3%)
Corynebacterium accolens	1 (1%)	-	-	-	-	1 (1%)
Clostridioides difficile	-	-	-	-	3 (100%)	3 (2%)
Citrobacter freundii	1 (1%)	-	1 (4%)	-	-	2 (1%)
Comamonas kerstersii	1 (1%)	-	-	-	-	1 (1%)
Citrobacter koseri	3 (3%)	-	1 (4%)	-	-	4 (2%)
Corynebacterium striatum	1 (1%)	-	-	-	-	1 (1%)
Corynebacterium tuberculostearicum	-	1 (3%)	-	-	-	1 (1%)
Coagulase-negative Staphylococcus aureus	-	7 (24%)	-	-	-	7 (4%)
Enterobacter cloacae	3 (3%)	-	-	-	-	3 (2%)
Escherichia coli	3 (3%)	1 (3%)	4 (17%)	-	-	8 (5%)
Enterococcus faecalis	5 (4%)	7 (24%)	6 (25%)	1 (20%)	-	19 (11%)
Enterococcus faecium	-	-	1 (4%)	-	-	1 (1%)
Granulicatella adiacens	-	1 (3%)	-	-	-	1 (1%)
Haemophilus influenzae	2 (2%)	-	-	-	-	2 (1%)
Klebsiella aerogenes	11 (10%)	1 (3%)	4 (17%)	1 (20%)	-	17 (10%)
Klebsiella oxytoca	3 (3%)	-	-	-	-	3 (2%)
Klebsiella pneumoniae	7 (6%)	-	3 (12%)	-	-	10 (6%)
Klebsiella variicola	1 (1%)	1 (3%)	-	-	-	2 (1%)
Methicillin-resistant Staphylococcus aureus	6 (5%)	1 (3%)	-	2 (40%)	-	9 (5%)
Methicillin-sensitive Staphylococcus aureus	34 (30%)	5 (17%)	-	-	-	39 (22%)
Nocardia nova	1 (1%)	-	-	-	-	1 (1%)
Pseudomonas aeruginosa	5 (4%)	-	3 (12%)	-	-	8 (5%)
Proteus mirabilis	1 (1%)	-	-	-	-	1 (1%)
Stenotrophomonas maltophilia	3 (3%)	-	-	-	-	3 (2%)
Serratia marcescens	8 (7%)	2 (7%)	1 (4%)	-	-	11 (6%)
Streptococcus milleri	3 (3%)	1 (3%)	-	1 (20%)	-	5 (3%)
Streptococcus pneumoniae	2 (2%)	-	-	-	-	2 (1%)
Streptococcus pseudopneumoniae	1 (1%)	-	-	-	-	1 (1%)
Streptococcus viridans	2 (2%)	-	-	-	-	2 (1%)

The most common type of infection was VAP, which was demonstrated by culture-confirmed infection in 55% (69/126) patients, with an additional 7 patients who received empiric therapy for VAP but never had positive lower respiratory cultures. *S. aureus* was isolated in 40 cases (34 MSSA and 6 MRSA), *K. aerogenes* in 11, and *S. marcescens* in 8 (Figure 2). Many patients had multiple pathogenic bacteria cultured from their lower respiratory tract: 4 patients had a total of 4 different species, 7 patients had 3 different species, and 18 patients had 2 species isolated. Notably, in 11 patients, organisms were repeatedly cultured from the lower respiratory tract for greater than 2 weeks despite appropriate antibiotic therapy. This included 1 patient who had MSSA continuously cultured from his lower respiratory tract for 3 months.

Bacteremia was the next most common type of infection, which affected 20% (25/126) of patients and caused a total of 29 unique infections, with 74% (22/29) of these taken from central venous catheters. The most common organisms were gram-positive bacteria with CoNS and *E. faecalis* as the most frequently isolated species causing 7 bloodstream infections each (Figure 2, Table 3). Four patients had recurrent bacteremia with different species including 1 patient with 3 different species isolated and 2 patients who had 2 distinct species.

Urinary tract infections were observed in 17% (21/126) of patients and were caused by 9 different bacterial species, 7 of which were Gram-negative rods. Urinary catheters were present in all patients for whom we have data (catheter presence/absence data was missing for 2 patients), and 15 patients met NHSN criteria for CAUTI (of note, 3 patients with UTIs not meeting criteria were diagnosed while on ECLS, and because normothermia is targeted with cooling of the circuit, the NHSN-required fever threshold of >38°C may be confounded in these cases). *E. faecalis* was the most frequently isolated organism with 6 infections, and *E. coli* was the next most common with 4 infections. Only 3 different patients had multiple UTIs, and all were limited to 2 infections. There were 5 different wound infections, 3 of which were infected tracheostomy sites which grew the same organism that was present in a concurrent VAP (Figure 2).There were also 3 patients with *C. difficile* colitis.

### Antibiotic Usage

**Figure 3.**
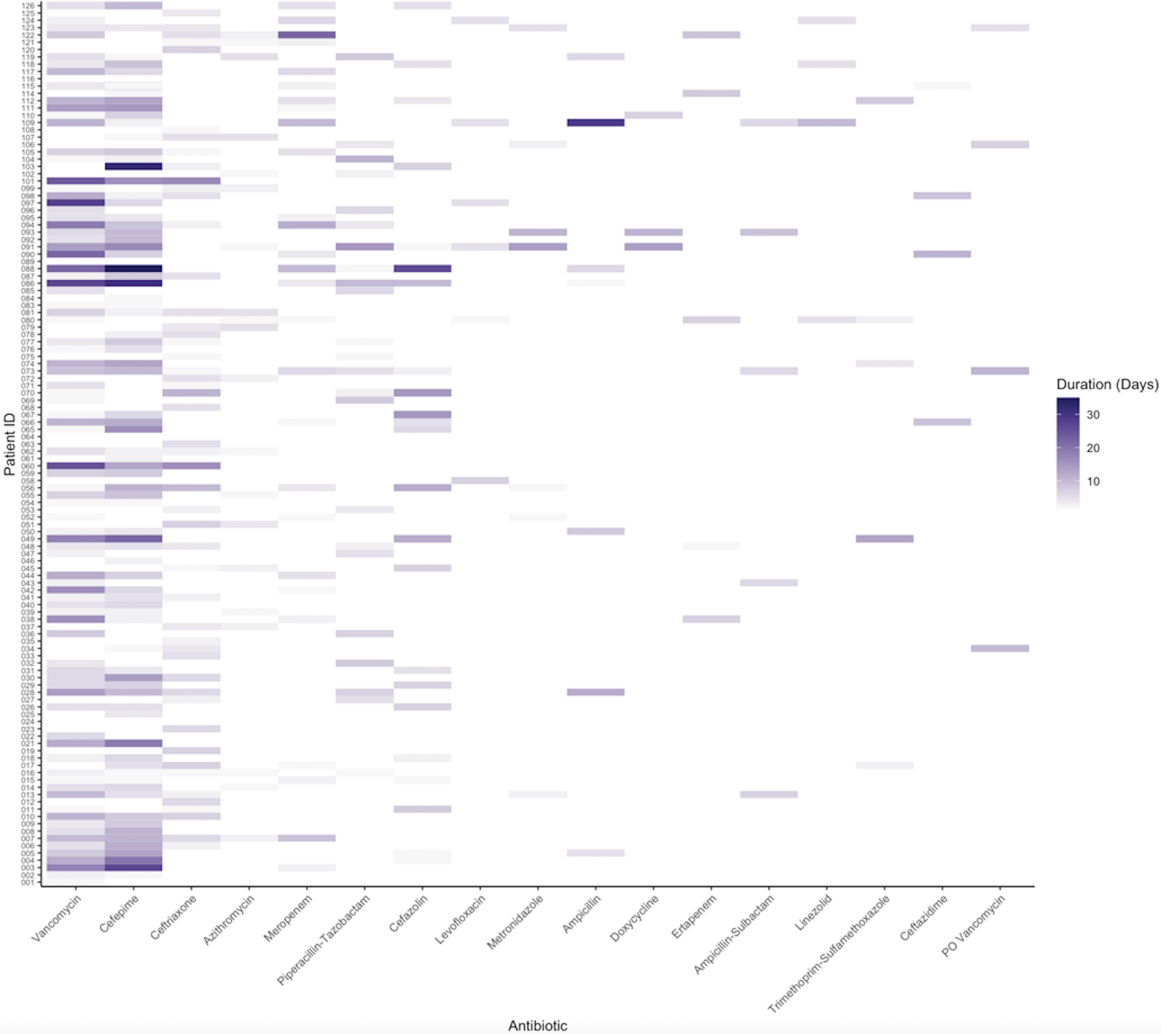
Heat map showing antibiotic exposures per patient. Each row in the y-axis corresponds to an individual patient, while each column in the x-axis corresponds to the antibiotic listed. The intensity of the coloration is relative to the number of days that a given antibiotic was administered. The antibiotics are listed in order of most frequently administered to least, and only those given to at least 5 patients are included in the figure.

In total, 97% (122/126) of the patients in this study population were exposed to at least one antibiotic during their hospital stay, and of those who received antibiotics, the average number of distinct antibiotics was 3.7 (st dev 1.8, range 1-9). The most common antibiotics given were vancomycin (78%) and cefepime (67%), followed by ceftriaxone and azithromycin (Figure 3). Of note, azithromycin was prescribed for a mean of only 2 DOT per patient (SD 1.3) (Online Appendix B), and was overwhelmingly prescribed in the context of empiric treatment for community acquired pneumonia prior to confirmation of Sars-CoV-2 infection. The median antibiotic DOT for all patients was 15 (IQR, 8-26.5). The median DOT for patients who had a culture confirmed SBI was 22.0 (IQR, 12-34) and 9.0 DOT (IQR, 6-13) for those without confirmed SBIs (*P* ≤ 0.0001). On average, a patient was exposed to 0.9 ± 0.6 antibiotics per day of hospitalization (including time on the acute care service after leaving the ICU).

## Discussion

Over the course of 2020, the spread of the SARS-CoV-2 virus resulted in a global pandemic with cases exceeding 100 million as of March 2021.^19^ The clinical syndrome of COVID-19 is widely variable and ranges from mild upper respiratory symptoms to severe ARDS requiring prolonged support with mechanical ventilation. To date, many studies investigating secondary bacterial infections in COVID-19 patients have relied on cohorts that include these heterogeneous populations of patients.^11^ Some have included both non-critically and critically ill patients,^20^ while others have focused on only critically ill patients but have not distinguished between intubated and non-intubated populations. Several meta-analyses have suggested low rates of secondary bacterial infections when compared to other viral pandemics, which has led to calls for a conservative antibiotic prescription strategy.^11,16,21,22^ In contrast, studies which focused specifically on the rates of VAP in COVID-19 have identified higher rates when compared to ventilated patients without COVID-19,^23^ with VAP rates of up to 86% in patients requiring ECLS.^24^ Other studies looking at central line associated infections have demonstrated higher rates during the pandemic compared to the preceding year.^25,26^

To help address this knowledge gap, we focused specifically on patients who were most severely affected by COVID-19 and required mechanical ventilation. With this strategy, we aimed to characterize the complete catalogue of secondary bacterial infections in COVID-19 ARDS as well as the antibiotic prescription practices of the clinical care teams taking care of these patients within this hospital system. In this cohort of mechanically ventilated patients with COVID-19, over 60% developed a secondary bacterial infection. Notably, 94% (164/174) of the culture-confirmed infections occurred after intubation. While this rate of infection is high, it is not surprising given the prolonged duration of mechanical ventilation and hospital stay in this cohort. It is also consistent with other studies which have primarily focused on the rates of VAP in patients with COVID-19.^23,24^ Many patients in this cohort suffered from multiple unique infections, including 31 patients who had VAP caused by multiple distinct bacterial species during their hospital course.

We notably had several patients who had the same pathogenic bacteria cultured from the respiratory tract over a prolonged time period despite appropriate therapy, leading to heavy antibiotic exposure in these individuals. Distinguishing between active infection and colonization in these patients is challenging due to the persistent but intermittent inflammatory state seen in patients with COVID-19, which is exacerbated by immunosuppression in sepsis which may predispose patients to colonization.^27,28^ Quantitative cultures, often obtained via bronchoscopy with bronchoalveolar lavage, may help improve specificity in VAP diagnosis. However, this can be prohibitively challenging when there is concern about generation of aerosols with a novel pathogen in resource-limited settings and overwhelmed health care systems during outbreaks. Further, prospective studies that specifically investigate antibiotic de-escalation strategies in these patients are warranted.

With regard to the bacterial pathogens isolated in SBIs, we found a similar composition of organisms to other studies of nosocomial ICU infections.^29^ *S. aureus* was the most common pathogen isolated in this investigation, consistent with other reports on SBIs in patients with COVID-19.^11,12^ Notably only 18.7% of these isolates displayed methicillin resistance. The rate of antibiotic prescribing in this cohort was very high, with 97% receiving at least one antibiotic despite 39% of patients not demonstrating a microbiologically confirmed SBI. This is consistent with findings from a previous study which reported that 80% of patients received an antimicrobial at some point during hospitalization regardless of culture results.^30^

Most patients were exposed to multiple different classes of antibiotics over the course of their hospital stay. The use of broad spectrum coverage was frequently employed, with cefepime (67%) and vancomycin (78%) being the most frequently prescribed antibiotics with an average duration of 8 days and 7 days, respectively. This likely reflects the clinical teams' inclination to prescribe an empiric course of antimicrobials for VAP, taking into consideration the critical illness of COVID-19 patients and the difficulty in differentiating bacterial infections from other clinical entities. Given the low prevalence of methicillin-resistant *S. aureus* (MRSA) in this cohort, the widespread use of vancomycin presents an opportunity for diagnostic and antimicrobial stewardship. Recent literature has highlighted MRSA nasal surveillance as a valuable screening tool to streamline vancomycin utilization given its high specificity and negative predictive value to rule out MRSA pneumonia.^31^. Empiric coverage for community acquired pneumonia was also common in our cohort, although only 7 patients had bacterial pulmonary infections at presentation. Interestingly, despite the high burden of antibiotic exposure there were only 3 *C. difficile* infections in the cohort.

The overall burden of antibiotic exposure in these patients was undeniably large, but in light of the high rate of SBIs, the wide prevalence of VAPs, the abundance of both Gram-negative rods and Gram-positive cocci isolated from multiple sites, and the low rate of *C. difficile*, we feel that the antibiosis strategies taken by the care teams during the study period were reasonable given the illness severity and diagnostic uncertainty in this cohort. Furthermore, patients who did not acquire SBIs had significantly fewer antibiotic DOT compared to those who did (9.0 vs 22.0, p = 0.0001), indicating that clinicians may have used culture data to guide prescription practices. We acknowledge that our findings differ from meta-analyses which have shown considerably lower rates of SBIs in patients with COVID-19. However, to our knowledge this is the first study fully cataloguing all SBIs in a specific cohort of mechanically ventilated COVID-19 patients, and as such, we expect our results to differ from studies which included heterogeneous populations. Indeed, our results are concordant with other studies which investigated the incidence of VAP in patients with COVID-19. There are, however, likely opportunities for critical care teams to partner with local stewardship champions to re-integrate established antimicrobial stewardship principles and discover new ones. Key areas to focus on include technology, diagnostics and guideline development emphasizing the maintenance of good infection control, surveillance for healthcare associated infections, and collating local data to promote appropriate antimicrobial usage to ultimately reduce the emergence of antimicrobial resistance.

Our study has several limitations. First, while the data acquisition occurred from 3 separate institutions, all were located within the same city, which may cause a geographic bias with respect to COVID-19 burden and the organisms isolated, potentially limiting generalizability. Second, there was not a standardized approach to sampling of lower respiratory tract isolates with a mix of bronchoalveolar lavages and tracheal aspirates used to inform clinical decision making. In many instances, patients underwent both of these modalities over the course of their hospital stay. As quantitative culture techniques were not uniformly employed, it is difficult to compare lower respiratory tract cultures across patients. Subsequently, VAP may have been overdiagnosed in this cohort. Relatedly, the retrospective nature of the study made us reliant on documentation in the medical record to determine if the clinical teams considered a given isolate a true infection or not. We acknowledge that there is potential misclassification of some isolates as true infection, colonization, or contaminant, but part of our aim was to elucidate the on-the-ground prescribing practices of the critical care teams and we believe this study is reflective of that. In addition, vancomycin use may be undercounted in this study, especially in patients with renal insufficiency who may not require daily dosing.

Our study is designed to highlight the challenges surrounding the diagnosis and management of secondary bacterial infections in severely ill patients affected by COVID-19. As both this study and the literature to date have shown that rates of bacterial co-infection and SBIs are relatively low in early COVID-19, especially among the non-critically ill, non-intubated population, we advocate for conservative use of antibiotic therapy guided by microbiology data in these groups. Considering the wide spectrum of clinical phenotypes in COVID-19 and the high rate of SBIs in this cohort, however, broad spectrum antimicrobials should be incorporated as part of the empiric treatment strategy for patients who are mechanically ventilated when sepsis is suspected. Yet, we advocate that their use is regularly re-evaluated for discontinuation or de-escalation, especially given the long durations of critical illness, prolonged positive respiratory cultures, and high antibiotic exposures noted in this cohort. We acknowledge the difficult tasks of clinicians caring for critically ill patients with COVID-19 and this study highlights the high risk of SBIs as well as the challenges in differentiating bacterial infection from COVID-19-related inflammation.

## Supplemental Material

Supplemental Material, sj-pdf-1-jic-10.1177_08850666211021745 - Characterization of Secondary Bacterial Infections and Antibiotic Use in Mechanically Ventilated Patients With COVID-19 Induced Acute Respiratory Distress Syndrome Click here for additional data file. Supplemental Material, sj-pdf-1-jic-10.1177_08850666211021745 for Characterization of Secondary Bacterial Infections and Antibiotic Use in Mechanically Ventilated Patients With COVID-19 Induced Acute Respiratory Distress Syndrome by Erik Risa, David Roach, Jehan Z. Budak, Christopher Hebert, Jeannie D. Chan, Nandita S. Mani, Chloe Bryson-Cahn, James Town and Nicholas J. Johnson in Journal of Intensive Care Medicine

Supplemental Material, sj-pdf-2-jic-10.1177_08850666211021745 - Characterization of Secondary Bacterial Infections and Antibiotic Use in Mechanically Ventilated Patients With COVID-19 Induced Acute Respiratory Distress Syndrome Click here for additional data file. Supplemental Material, sj-pdf-2-jic-10.1177_08850666211021745 for Characterization of Secondary Bacterial Infections and Antibiotic Use in Mechanically Ventilated Patients With COVID-19 Induced Acute Respiratory Distress Syndrome by Erik Risa, David Roach, Jehan Z. Budak, Christopher Hebert, Jeannie D. Chan, Nandita S. Mani, Chloe Bryson-Cahn, James Town and Nicholas J. Johnson in Journal of Intensive Care Medicine
