# Supplementary material for: Characterization of Secondary Bacterial Infections and Antibiotic Use in Mechanically Ventilated Patients With COVID-19 Induced Acute Respiratory Distress Syndrome
Source: J Intensive Care Med. 2021 Aug 10;36(10):1167–75. doi: 10.1177/08850666211021745 (PMC8358424; doi:10.1177/08850666211021745)
Supplement: Supplemental Material, sj-pdf-1-jic-10.1177_08850666211021745 - Characterization of Secondary Bacterial Infections and Antibiotic Use in Mechanically Ventilated Patients With COVID-19 Induced Acute Respiratory Distress Syndrome [file sj-pdf-1-jic-10.1177_08850666211021745.pdf]

Appendix A: Positive cultures within first 48 hours of admission

Patient	Infection
1	E. coli (urine)
2	E. coli (urine), E. coli (ET aspirate)
3	E. coli (urine)
4	E. faecalis (CVC)
5	MRSA (ET aspirate), E. coli (ET aspirate)
6	MSSA (sputum)
7	MSSA (sputum)
8	S. pneumoniae (ET aspirate)
9	S. pneumoniae (ET aspirate), H. influenzae
10	S. pneumoniae (ET aspirate), H. influenzae (ET aspirate), S. aureus (ET aspirate)
