# Supplementary material for: Characterization of Secondary Bacterial Infections and Antibiotic Use in Mechanically Ventilated Patients With COVID-19 Induced Acute Respiratory Distress Syndrome
Source: J Intensive Care Med. 2021 Aug 10;36(10):1167–75. doi: 10.1177/08850666211021745 (PMC8358424; doi:10.1177/08850666211021745)
Supplement: Supplemental Material, sj-pdf-2-jic-10.1177_08850666211021745 - Characterization of Secondary Bacterial Infections and Antibiotic Use in Mechanically Ventilated Patients With COVID-19 Induced Acute Respiratory Distress Syndrome [file sj-pdf-2-jic-10.1177_08850666211021745.pdf]

Appendix B: Antibiotic Days of Therapy and Exposures

Antibiotic	Days of Therapy Mean (SD)	Count (%)
Vancomycin	7 (6.3)	97 (77.6%)
Cefepime	8.4 (7.2)	84 (67.2%)
Ceftriaxone	3.9 (3.2)	66 (52.8%)
Azithromycin	2 (1.3)	41 (32.8%)
Meropenem	4.9 (4.3)	30 (24.0%)
Piperacillin-tazobactam	4.8 (3.5)	28 (22.4%)
Cefazolin	6.4 (5.7)	27 (21.6%)
Levofloxacin	3.6 (2.3)	9 (7.2%)
Metronidazole	5.1 (4.8)	8 (6.4%)
Ampicillin	9.7 (9)	7 (5.6%)
Ertapenem	5.7 (3.3)	6 (4.8%)
Doxycycline	5.8 (5.7)	6 (4.8%)
Trimethoprim-Sulfamethoxazole	15.3 (22.7)	6 (4.8%)
Ceftazidime	6.6 (4.3)	5 (4.0%)
Ampicillin-Sulbactam	6.8 (1.3)	5 (4.0%)
Linezolid	5.4 (2.9)	5 (4.0%)
PO Vancomycin	8.2 (2.8)	4 (3.2%)
Amoxicillin-Clavulanate	2.7 (2.1)	3 (2.4%)
Ciprofloxacin	9 (13)	3 (2.4%)
Ceftaroline	7 (4.6)	3 (2.4%)
Clindamycin	2	2 (1.6%)
Aztreonam	5.5	2 (1.6%)
Amoxicillin	2	1 (0.8%)
Gentamicin	1	1 (0.8%)
Penicillin-V	17	1 (0.8%)
Ceftolozane-Tazobactam	5	1 (0.8%)
Amikacin	1	1 (0.8%)
Imipenem-Cilastatin	31	1 (0.8%)
Avibactam-Ceftazidime	1	1 (0.8%)
Cephalexin	4	1 (0.8%)
Daptomycin	2	1 (0.8%)
